# Proteolytic inactivation of nuclear alarmin high-mobility group box 1 by complement protease C1s during apoptosis

**DOI:** 10.1038/cddiscovery.2016.69

**Published:** 2016-09-12

**Authors:** J G Yeo, J Leong, T Arkachaisri, Y Cai, B H D Teo, J H T Tan, L Das, J Lu

**Affiliations:** 1 Department of Microbiology and Immunology, Immunology Program, Yong Loo Lin School of Medicine, National University of Singapore, Singapore; 2 Division of Medicine, KK Women’s and Children’s Hospital, Singapore; 3 SingHealth Translational Immunology and Inflammation Center, Singapore Health Services, Singapore; 4 Rheumatology and Immunology Service, Department of Pediatric Subspecialities, KK Women’s and Children’s Hospital, Singapore; 5 Duke-National University of Singapore Medical School, Singapore

## Abstract

Effective clearance of apoptotic cells by phagocytes prevents the release of intracellular alarmins and manifestation of autoimmunity. This prompt efferocytosis is complemented by intracellular proteolytic degradation that occurs within the apoptotic cells and in the efferosome of the phagocytes. Although the role of extracellular proteases in apoptotic cells clearance is unknown, the strong association of congenital C1s deficiency with Systemic Lupus Erythematosus highlights the protective nature that this extracellular protease has against autoimmunity. The archetypical role of serine protease C1s as the catalytic arm of C1 complex (C1qC1r_2_C1s_2_) involve in the propagation of the classical complement pathway could not provide the biological basis for this association. However, a recent observation of the ability of C1 complex to cleave a spectrum of intracellular cryptic targets exposed during apoptosis provides a valuable insight to the underlying protective mechanism. High-mobility group box 1 (HMGB1), an intracellular alarmin that is capable of inducing the formation of antinuclear autoantibodies and causes lupus-like conditions in mice, is identified as a novel potential target by bioinformatics analysis. This is verified experimentally with C1s, both in its purified and physiological form as C1 complex, cleaving HMGB1 into defined fragments of 19 and 12 kDa. This cleavage diminishes HMGB1 ability to enhance lipopolysaccharide mediated pro-inflammatory cytokines production from monocytes, macrophages and dendritic cells. Further mass spectrometric analysis of the C1 complex treated apoptotic cellular proteins demonstrated additional C1s substrates and revealed the complementary role of C1s in apoptotic cells clearance through the proteolytic cleavage of intracellular alarmins and autoantigens. C1 complex may have evolved as, besides the bacteriolytic arm of antibodies in which it activates the complement cascade, a tissue renewal mechanism that reduces the immunogenicity of apoptotic tissue debris and decreases the likelihood of autoimmunity.

## Introduction

Systemic lupus erythematosus (SLE) is an autoimmune disease with protean clinical presentations and its etiology remains partially defined.^1^ However, two pathological hallmarks of the disease have been established including the excessive production of interferon-*α* (IFN-*α*)^2^ and formation of antinuclear autoantibodies.^3^ These antinuclear autoantibodies typically surge in SLE patients before disease flare and have important prognostic value.^4,5^ Concurrently in active disease, the circulatory level of nuclear autoantigens, typically nucleosome either from an elevated cell death or impaired clearance also increases.^3,6^ These two factors in combination will lead to the formation and deposition of injurious immune complexes in tissues. In addition, these immune complexes formed between SLE autoantibodies and autoantigens can induce dendritic cells (DC) IFN-*α* production.^7^

SLE is a polygenic disease with 40–50 susceptibility genes identified. However, the majority are not lupus-specific and exhibit a small effect size (with odds ratio <2.0)^8,9^ with the exception of deficiencies in Trex1, C1q, C1r/C1s, and C4 which have a higher odds ratio of 5 to 25.^10^ Trex1 is a 3ʹ-5ʹ exonuclease which degrades nicked double-stranded DNA (dsDNA), created by the serine protease granzyme A.^11^ *In vivo*, a nuclease-inactive Trex1 variant causes antinuclear autoantibodies formation and lupus-like disease suggesting that impaired clearance of nuclear DNA contributes to its pathogenesis.^12^ The remaining 4 implicated proteins are intimately related in the formation of C1 complex (C1qC1r_2_C1s_2_) which, upon C1q binding to ligands, are activated to cleave C4, thereby initiating the complement classical pathway.^13^

How C1r/C1s deficiency triggers SLE-like conditions has not been investigated mechanistically. In contrast, C1q’s protective role against SLE has been extensively studied and four potential mechanisms have emerged. First, C1q binds to apoptotic cells that can opsonize cell debris for effective efferocytosis.^14–16^ Second, C1q-bound apoptotic cells induce immunosuppressive properties in phagocytes.^17^ Third, C1q induces tolerogenic properties in DC during development.^18^ Finally, C1q was shown to inhibit IFN-*α* induction by the lupus immune complexes.^19,20^ Both Trex1 and C1q appear to contribute to immune tolerance by limiting the leakage of intracellular autoantigens and alarmins and hence reducing the activation of autoreactive lymphocytes.^21^

To date, 22 C1r/C1s deficiency cases have been reported.^22–27^ Hereditary deficiencies of C1r and C1s tend to occur concomitantly.^28^ Within the complement system, C1s is a highly specific protease that cleaves C4, C2 and C1-inhibitor (C1-INH).^13,29^ Binding of C1q to its ligands activates C1r with C1r then cleaving C1s specifically to cause its activation. Non-complement C1s substrates including insulin-like growth factor binding protein 5 (IGFBP5),^30^ major histocompatibility complex class I subunits,^31^ and low-density lipoprotein receptor-related protein 6 (LPR6)^32^ have been described. However, cleavage of these non-complement and complement proteins by C1s does not provide a biological plausible explanation to its protective role against the development of autoimmunity.

Through bioinformatics, a broad spectrum of intracellular proteins were predicted to contain C1s cleavage sites despite their perceived inaccessibility in live cells.^33^ The significance of this finding only became apparent recently when we observed the prominent binding of C1q to the nucleolus of apoptotic cells and the resultant degradation of the nucleolar proteins, nucleophosmin 1 (NPM1) and nucleolin, in the copresence of the protease C1s with C1q found in C1 complex.^34^ Both of these proteins were predicted to contain C1s cleavage sites.^34^

The nuclear protein HMGB1 is a novel substrate that has also been predicted to contain C1s cleavage sites. HMGB1 is a DNA-binding nuclear protein with defined roles in DNA bending and can be released during cell apoptosis or activation.^35,36^ Extracellular HMGB1 has a wide range of immunological activities such as induction of macrophages/monocytes cytokine production and DC maturation.^35^ It is also involved in the pathogenesis of autoimmune diseases.^36^ Specifically, HMGB1 containing nucleosome induces antinuclear autoantibodies formation and SLE-like conditions in mice.^37^ One mechanism by which it activates monocytes requires its binding to lipopolysaccharide (LPS) with subsequent transfer of the LPS to CD14 to enhance toll-like receptor 4 (TLR4)-mediated tumor necrosis factor-*α* (TNF-*α*) production.^38^ In the present study, we examine whether C1s actually cleaves HMGB1 and inactivates the pro-inflammatory activities of this alarmin protein.

## Results

### HMGB1 contains three potential C1s cleavage sites

**Figure 1:**
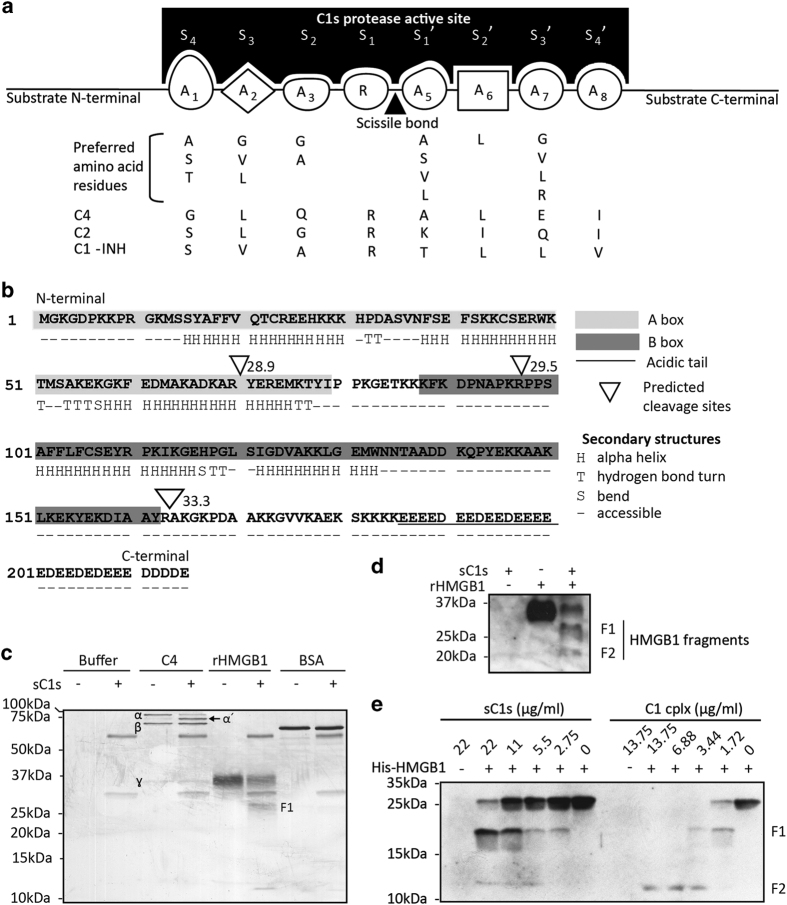
HMGB1 is cleavable by C1s. (**a**) The PoPS model for predicting C1s substrate sequences. The C1s protease active site contains eight contiguous pockets or subsites each having a unique structural and chemical property and preferred specific amino acid residues (Single letter amino acid code is used). These amino acid residues were experimentally determined previously.^33^ The subsites display different importance with varying specificity except for the A_4_ position where an arginine, which is conserved in C4, C2 and C1-INH, is considered an essential residue. Position of the `scissile bond’ is indicated with triangle. (**b**) Predicted C1s cleavage sites on HMGB1. The HMGB1 sequence is presented and the A box and B-box are shaded. The C-terminal acidic tail is underlined. Where secondary structures are predicted, these are indicated under the sequence. `-’, lack of secondary structure. The 3 inverted triangles indicate predicted C1s cleavage sites with PoPS scores (Arg70, Arg97 and Arg163). (**c**) Purified rHMGB1 was cleaved by purified sC1s. RHMGB1 produced in mouse myeloma cells (10 *μ*g/ml), serum C4 (10 *μ*g/ml), and BSA (5 *μ*g/ml) were incubated overnight at 37 °C with activated sC1s (11 *μ*g/ml). The samples were separated by SDS-PAGE on 12.5% (w/v) gels under reducing conditions and visualized by silver staining. The three C4 subunit chains (*α*, *β* and *γ*) are labeled. The cleaved C4 *α* chain was labeled *α*’. After C1 digestion of rHMGB1, a fragment was generated (F1). (**d**) Two HMGB1 fragments were detected after C1s digestion by western blotting (F1 and F2). Western blotting was performed using a rabbit polyclonal anti-HMGB1 antibody. (**e**) C1 complex (C1 cplx) cleaved HMGB1 more effectively than C1s but both generated two similar fragments. In this experiment, His-HMGB1 expressed in bacteria *E. coli* (20 *μ*g/ml) was incubated overnight at 37 °C with C1 complex and activated sC1s at the concentrations indicated. The reactions were separated by SDS-PAGE on 15% (w/v) gels under reducing conditions and western blotting was performed using the polyclonal rabbit anti-HMGB1 antibody. F1 and F2 marked the HMGB1 fragments after cleavage.

Firstly, a C1s substrate prediction model was constructed using the prediction of protease specificity (PoPS) software (Figure 1a).^33,39^ With this model, three potential C1s cleavage sites were predicted in HMGB1 with different PoPS scores with a higher score indicative of a greater likelihood of cleavage (Figure 1b). HMGB1 is a 215 amino acid protein consisting of 2 DNA-binding domains (A and B boxes) and a C-terminal acidic tail.^40^ The predicted cleavage site at Arg70 is within the A box domain but is part of a secondary helix structure which could hinder C1s access. The sites at Arg97 and Arg163 are located within exposed regions as predicted by PoPS.^39,41^ The prediction of three C1s cleavage sites in HMGB1, a pro-inflammatory nuclear alarmin associated with SLE-like conditions,^37^ led us to examine whether C1s cleaves HMGB1 and inactivates its pro-inflammatory activities.

### Both C1s and the C1 complex cleave HMGB1

Recombinant HMGB1 (rHMGB1) and as controls, complement C4 and bovine serum albumin (BSA), were treated with purified human serum C1s (sC1s). C1s cleaved only the *α* chain of C4, its natural substrate, but did not cleave BSA (Figure 1c). However, HMGB1 was partially cleaved giving rise to a 25-kDa fragment as detected by silver staining (Figure 1c). By western blotting, an additional HMGB1 fragment of ~20 kDa was also detected (Figure 1d). Based on its sequence, HMGB1 is predicted as a 25 kDa protein. However, rHMGB1 produced from myeloma cells exhibited a higher molecular weight of ~35 kDa. Therefore, the actual C1s cleavage sites on rHMGB1 could not be deduced based on the fragment sizes. Polyhistidine-tagged HMGB1 (His-HMGB1) expressed in bacteria had a molecular weight of 25 kDa and was therefore used for this purpose.

C1s cleaved His-HMGB1 in a dose-dependent manner (2.75–22 *μ*g/ml; Figure 1e). At 11 *μ*g/ml of C1s, His-HMGB1 cleavage was prominent but remained incomplete. At 22 *μ*g/ml, C1s caused near-complete His-HMGB1 cleavage. Two fragments of ~12 and 19 kDa were generated. The 12-kDa fragment corresponds in size to an N-terminal HMGB1 fragment with a cleavage at Arg97 (11.4-kDa) and the 19-kDa fragment will correspond to cleavage at Arg163 (18.9 kDa).

As C1s occurs physiologically as C1 complex, His-HMGB1 was similarly incubated with C1. C1 degraded His-HMGB1 more effectively (Figure 1e). At a concentration of 3.44 *μ*g/ml, His-HMGB1 was completely degraded. This was much more potent than C1s alone as His-HMGB1 digestion remained partial at 22 *μ*g/ml of the protease. C1 cleavage of His-HMGB1 also generated fragments of 12 and 19 kDa.

### Apoptotic HMGB1 is degraded by C1 complex

**Figure 2:**
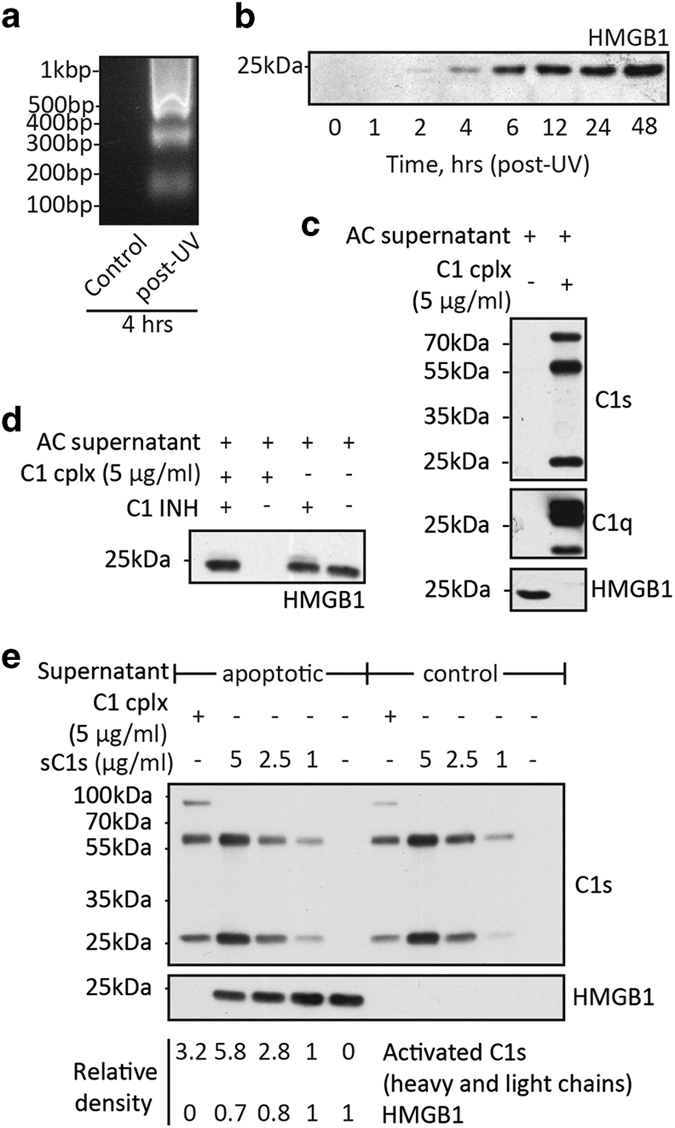
C1 and C1s cleavage of HMGB1 released from apoptotic cells. (**a**) DNA laddering of UV-irradiated Jurkat cells. Cells were, after UV irradiation, cultured for 4 h in serum-free medium (post UV). As control, cells were cultured for 4 h without prior UV irradiation (control). Extracted DNA was examined on 1.2% (w/v) agarose gels and DNA was visualized using GelGreen (Biotium, Inc., Hayward, CA, USA) (**b**) HMGB1 release from apoptotic cells. After UV irradiation, Jurkat cells were cultured for a series of time periods up to 48 h. After centrifugation, supernatants were harvested and subjected to SDS-PAGE on 12.5% (w/v) gels and western blotting using a rabbit polyclonal anti-HMGB1 antibody. (**c**) C1 degradation of HMGB1 released from apoptotic cells. In this experiment, Jurkat cells were harvested 2.5 h after UV irradiation and, after mixing, divided in 490-*μ*l aliquots. C1 (240 *μ*g/ml) was added to a final concentration of 5 *μ*g/ml (10.4 *μ*l) and incubated for 30 min at 37 °C. After centrifugation, supernatants were separated by SDS-PAGE on 12.5% gels and then analyzed by western blotting using goat anti-C1q and rabbit anti-C1s and anti-HMGB1 antibodies. (**d**) C1 cleavage of HMGB1 was inhibited by C1-INH. Apoptotic cells were incubated with C1 for 30 min at 37 °C in the presence or absence of C1-INH (105 *μ*g/ml). As controls, apoptotic cells were incubated with C1-INH only. After centrifugation, supernatants were subjected to SDS-PAGE on 12.5% (w/v) gels and western blotting using the rabbit anti-HMGB1 antibody. (**e**) Comparison of C1 with C1s in HMGB1 cleavage during apoptosis. As in (**c**), apoptotic cells harvested 2.5 h after UV irradiation were incubated for 30 min at 37 °C with C1 (5 *μ*g/ml) or sC1s (1.0, 2.5 or 5.0 *μ*g/ml). As controls, Jurkat cells were incubated with C1 or C1s without prior UV irradiation. After centrifugation, supernatants were subjected to SDS-PAGE on 12.5% gels and western blotting using the rabbit anti-HMGB1 antibody. Band intensities were quantified by densitometry. Values for C1s represent combined signals of the heavy and light chains in each lane. Values for HMGB1 were derived from the single band in each lane. Note the reduced HMGB1 intensities at 2.5 and 5.0 *μ*g/ml of sC1s. Apoptotic cells: AC.

HMGB1 released by apoptotic cells has been reported to cause the formation of antinuclear autoantibodies and SLE-like conditions in mice.^37^ We therefore asked whether apoptotic HMGB1 is cleavable by C1 or C1s. Jurkat cells were UV (ultra violet)-irradiated and apoptosis was evident 4 h later with detectable DNA laddering (Figure 2a). At 2 h post UV irradiation, HMGB1 was released and this steadily increased over time (Figure 2b).

To examine HMGB1 cleavage by C1 and C1s, Jurkat cells were allowed to undergo apoptosis for 2.5 h after UV irradiation and then incubated for 30 min at 37 °C with the proteases (C1 at 5 *μ*g/ml, sC1s at 1, 2.5 or 5 *μ*g/ml, or phosphate-buffered saline (PBS)). Supernatants were collected and HMGB1, C1s and C1q were examined by western blotting. HMGB1 was prominently detected in the supernatant without C1 treatment (Figure 2c). With C1-treated apoptotic cells, C1q and C1s were prominently detected in the supernatant with correspondingly diminished HMGB1 levels (Figure 2c). This showed that C1 effectively degraded apoptotic HMGB1. C1 proteases were involved as the observed HMGB1 degradation was effectively abrogated by C1-INH (Figure 2d). Therefore, C1 appears to be a potentially effective mechanism in the degradation of HMGB1 and possibly other intracellular proteins, which may otherwise be released by apoptotic cells as immunogenic alarmins or self-antigens.

C1s also degraded apoptotic HMGB1 in a dose-dependent manner (Figure 2e). However, compared with C1, C1s alone was much less effective. Apoptotic HMGB1 was completely degraded by C1 at the concentration of 5 *μ*g/ml, but a significant fraction of HMGB1 remained intact after treatment with the same concentration of C1s (Figure 2e). This was not surprising as C1s is a much more effective protease when present physiologically as C1 complex.^42^ Without UV irradiation, HMGB1 was not released from the Jurkat cells (Figure 2e).

### C1s cleavage of HMGB1 diminishes its synergy with LPS

**Figure 3:**
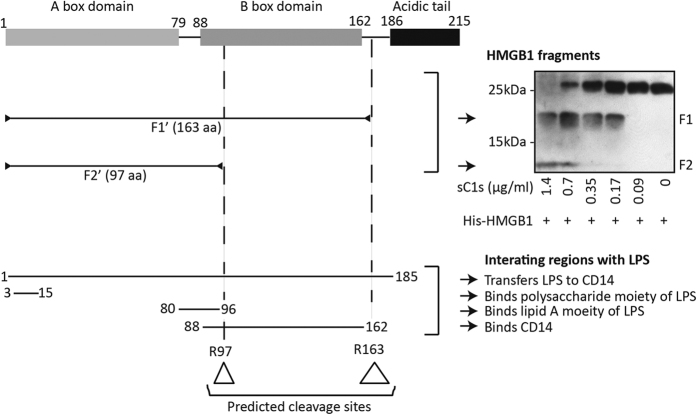
Alignment of HMGB1 fragments potentially generated after C1s cleavage and HMGB1 regions that interact with LPS and CD14. Based on the sizes of the two HMGB1 fragments after C1s digestion, that is, 19 and 12 kDa (right panel), the two likely cleavage sites are Arg163 (F1ʹ) and Arg97 (F2ʹ). In this western blotting experiment, bacteria-expressed His-HMGB1 was used. SC1s was used at reducing concentrations. The corresponding LPS and CD14 interactive regions in HMGB1 were aligned below. Note that, both C1s cleavage sites fall into bioactive regions of HMGB1 (triangles) and can potentially impair HMGB1 synergy with LPS.

HMGB1 synergizes with LPS in stimulating immune cells and this is dependent on the structural integrity of the protein.^38,43^ The LPS and CD14-binding sites on HMGB1 reside from the N terminus to residue 162 (Figure 3).^38,44^ C1s cleavage at Arg163 is expected to preserve this LPS-synergistic property in HMGB1. However, this pro-inflammatory activity is expected to be impaired if cleavage occurs at Arg97. There was a clear dose-dependent cleavage of HMGB1 by C1s (0.09–1.4 *μ*g/ml) and the two characteristic HMGB1 fragments were also clearly observed. We then proceeded to co-stimulate macrophages, DC and monocytes with C1s-digested HMGB1 and LPS.

### C1s inactivates the pro-inflammatory effect of HMGB1

**Figure 4:**
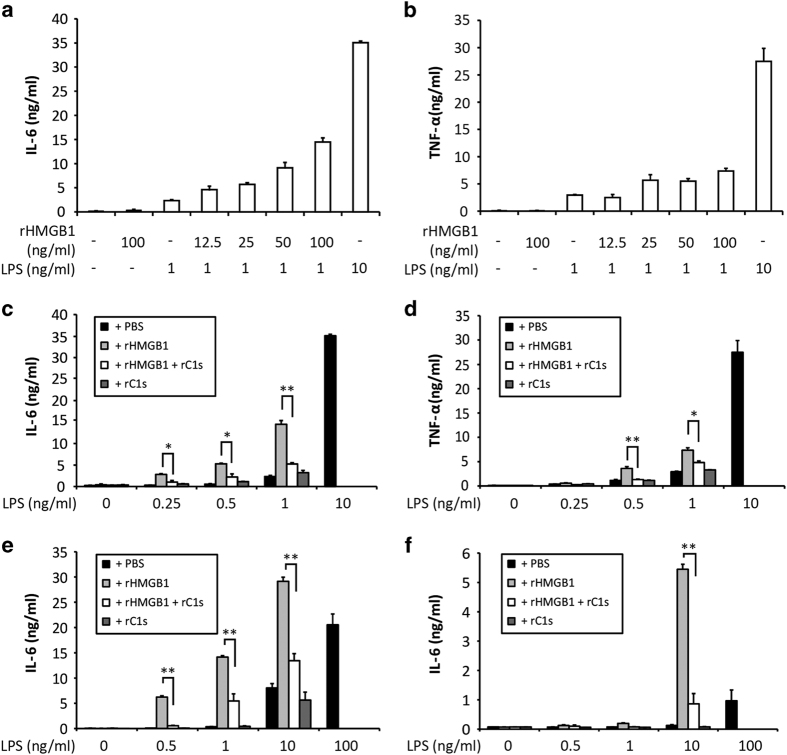
HMGB1 enables monocyte, macrophage and DC activation by suboptimal levels of LPS but this is inactivated with C1s digestion. Macrophages were stimulated for 20 h with LPS at 1 ng/ml and rHMGB1 was added at 12.5, 25, 50 and 100 ng/ml. As controls, macrophages were stimulated with LPS at 1 and 10 ng/ml without rHMGB1. Production of IL-6 (**a**) and TNF-*α* (**b**) was measured by ELISA. In another experiment, different concentrations of LPS (12.5–500 ng/ml) were pre-incubated for 20 h at 37 °C with rHMGB1 (5 *μ*g/ml) in the presence or absence of rC1s (2.75 *μ*g/ml). As controls, LPS was incubated with PBS or rC1s. These stimuli mixtures were used at 1/50 dilution to stimulate macrophages so that the final concentration of HMGB1 and rC1s in the culture are 100 and 55 ng/ml respectively. IL-6 (**c**) and TNF-*α* (**d**) production was determined by ELISA. DC (**e**) and monocytes (**f**) were similarly stimulated except that LPS concentrations were increased in the pre-incubation mixture (25–5000 ng/ml). IL-6 production was determined by ELISA. Experiments were performed in triplicates. Results were analyzed using Student’s 2-tailed unpaired *t*-test and presented as mean±S.D. *P<0.05, **P<0.01.

In this experiment, rHMGB1 and recombinant C1s (rC1s) were used to avoid contaminants of bacterial origin that might co-purify with His-HMGB1 (e.g., TLR ligands)^43^ and C1s contamination by serum LPS-binding protein. At 10 ng/ml, LPS induced both TNF-*α* and IL-6 from macrophages (Figure 4a and b). However, little was induced with LPS at 1 ng/ml. When LPS was incubated with rHMGB1 before macrophage stimulation, the otherwise suboptimal levels of LPS became competent in inducing the production of these cytokines, especially IL-6, in a rHMGB1 dose-dependent manner (12.5–100 ng/ml; Figure 4a). RHMGB1 also enhanced LPS induced TNF-*α* production albeit less prominent than IL-6 (Figure 4b). As a control, rHMGB1 alone was not able to induce macrophage TNF-*α* and IL-6 production.

RHMGB1 (5 *μ*g/ml) was then incubated with C1s (2.75 *μ*g/ml) for 20 h in the presence of three different concentrations of LPS (12.5, 25 and 50 ng/ml). These were diluted 50 folds to stimulate the macrophages. As controls, LPS was incubated with rHMGB1, C1s or PBS separately. Incubation with rHMGB1 enhanced LPS macrophage cytokines induction, especially IL-6 (Figure 4c and d). When C1s was present during rHMGB1 incubation with LPS, rHMGB1 cleavage occurred (data not shown) and the synergistic effect of rHMGB1 on LPS IL-6 induction was effectively diminished (Figure 4c). A similar trend was observed with respect to TNF-*α* induction (Figure 4d).

Similar experiments were performed using DC and monocytes. With DC, little IL-6 induction was observed at 0.5 to 1 ng/ml of LPS but incubation with rHMGB1 enabled it to induce IL-6 from these cells (Figure 4e). Similarly in monocytes, low level of IL-6 induced at a LPS concentration of 10 ng/ml was greatly enhanced by the presence of rHMGB1 (Figure 4f). Treatment with C1s reduced HMGB1 enhancing effect on LPS induced cytokine production (Figure 4e and f). The data collectively demonstrated a synergistic effect of rHMGB1 on LPS that enabled suboptimal levels of LPS to activate monocyte, macrophage and DC. These cells are important in orchestrating inflammation and adaptive immune responses. Subclinical levels of plasma LPS exists and appears to induce tolerogenic status.^45^ Apoptotic HMGB1 could prime this status into a pro-inflammatory state.

### C1 cleaves other proteins released by apoptotic cells

**Figure 5:**
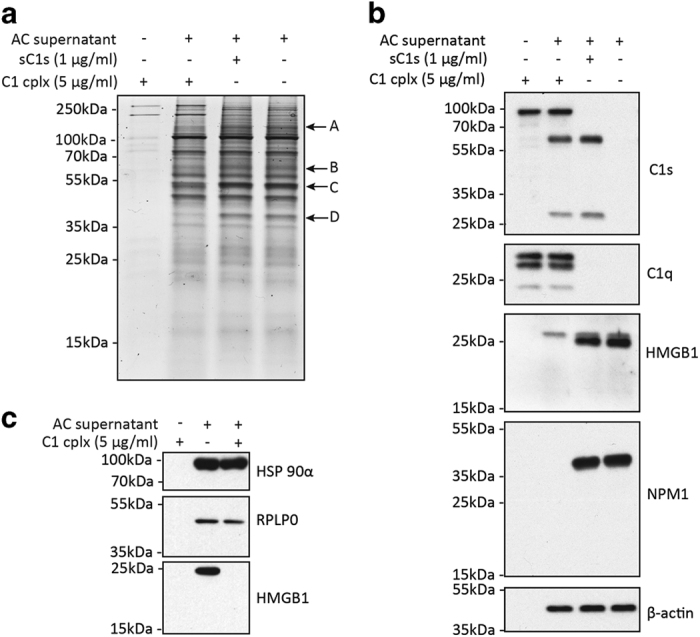
C1 cleaves multiple proteins released by apoptotic cells. (**a**) Visible protein profile released from apoptotic cells and their susceptibility to C1 or C1s degradation. UV-irradiated Jurkat cells were cultured for 2.5 h and then incubated with sC1s, C1 or PBS for 30 min at 37 °C. After centrifugation, supernatants were harvested and subjected to SDS-PAGE on 15% (w/v) gels. Proteins were visualized by Commassie blue staining. Band regions in the C1-treated sample that diminished compared with control sample (PBS-treated) are indicated with arrows and alphabets. Four band regions were identified: A (~100 kDa), B (~60 kDa), C (~50 kDa) and D (~40 kDa). In a separate gel, these four band regions in the control sample were excised for mass spectrometry analysis (Supplementary Table 1). (**b**) The separated proteins were also analyzed by western blotting where HMGB1, NPM1, *β*-actin and, as controls, C1s and C1q were detected. (**c**) C1 showed no cleavage of HSP90*α* and RPLP0. By mass spectrometry, HSP90*α* was detected in band A and RPLP0 was found in band C (Supplementary Table 1). Western blot detection of HSP90*α* and RPLP0 were done using rabbit polyclonal antibodies. HMGB1 was used as a control. AC: apoptotic cells.

At this point, we decided to view the global protein profile released by apoptotic cells by blue silver staining^46^ and the range of proteins that might be susceptible to C1 and C1s cleavage. By comparing the profiles with or without C1 or C1s treatment, four protein bands (A–D) of ~100, 60, 50 and 40 kDa, respectively, were markedly reduced with C1 treatment (Figure 5a). Although HMGB1 was expected to be degraded, there was no clear change at the 25-kDa region after C1 treatment. Furthermore, majority of the visible protein bands were not significantly affected by C1 treatment and the 30-min C1s digestion caused no significant changes in the protein profile (Figure 5a). Mass spectrometry analysis was performed with the four band regions which identified 5–7 candidate proteins in each region (Supplementary Table 1). NPM1 was present in band D.

To ascertain the extents to which HMGB1 and NPM1 in apoptotic supernatants might be cleaved by C1 proteases, these supernatants were subjected to western blotting. C1s was activated after C1 incubation with apoptotic cells as judged by the appearance of the 56-kDa heavy chain and 27-kDa light chain (Figure 2c and e, Figure 5b). C1q was clearly detected. The blots were probed for HMGB1, NPM1 and, as a control, *β*-actin. HMGB1 was nearly completely degraded by C1 and was also partially reduced after C1s treatment (Figure 5b). NPM1 was completely degraded by C1 but not C1s.

Heat shock protein 90 *α* (HSP90*α*) and 60S acidic ribosomal protein P0 (RPLP0) were detected in the 100-kDa band A and 50-kDa band C, respectively (Supplementary Table 1). Altogether with NPM1, these are three autoantigens present in SLE patients.^47–49^ We, therefore, examined whether HSP90*α* and RPLP0 were cleaved after C1 treatment by western blotting, but neither was cleaved unlike NPM1 and HMGB1 which were effectively cleaved (Figure 5c). These results suggest the substrate selectivity of C1 proteases. The exact protein species in the other three bands (A–C) that were also cleaved by C1 remain to be confirmed. Some proteins like HMGB1 may be cleaved but are not visible by blue silver staining due to lower abundance. Nonetheless, C1 proteases exhibit prominent yet selective cleavage of apoptotic cellular proteins.

### C1 cleaves selective autoantigens

At this point, a curious question was the extent to which the ratio of detectable autoantigens might be cleaved by C1. For this experiment, supernatant from apoptotic U937 cells were used because it was previously reported to contain abundant autoantigens and form immune complexes with SLE autoantibodies that potently induced IFN-*α* from DC.^7,50^ U937 cells were, after UV irradiation, cultured for 24 h in PBS and the supernatant was analyzed by western blotting using eight rheumatological patients’ sera (Supplementary Table 2).

**Figure 6:**
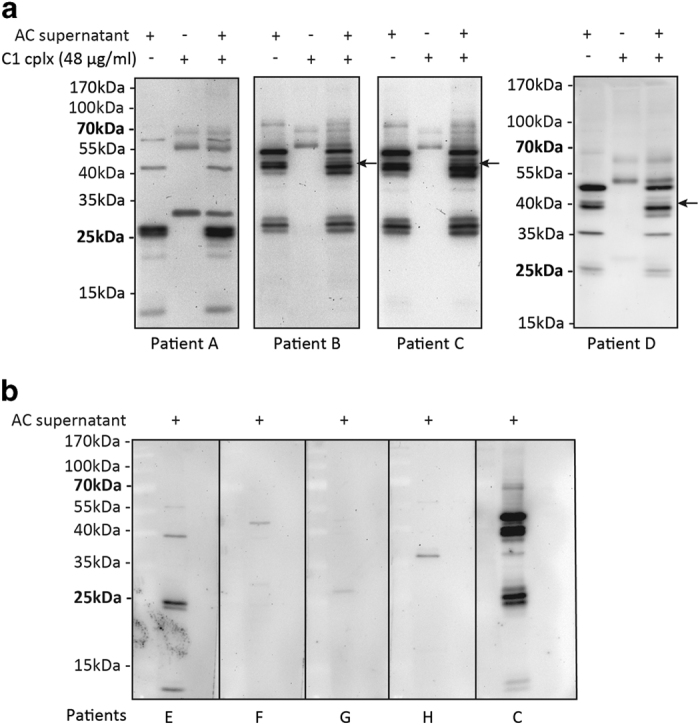
C1 cleavage of autoantigens released by apoptotic cells. U937 cells (5×10^7^/ml) in PBS were UV-irradiated. The supernatant was obtained after 24 h and incubated for 1 h with C1 (48 *μ*g/ml) or, as a control, with PBS. The supernatant samples were then separated by SDS-PAGE on 12.5% (w/v) gels and analyzed by western blotting with rheumatological patient sera at 1 : 5000 dilutions. Horseradish peroxidase-conjugated goat anti-human IgG Fc was used as secondary antibody and signals were visualized using the Bio-Rad MPS ChemiDoc imaging system. (**a**) Sera from four patients detected autoantigens in the apoptotic U937 supernatant. A 45-kDa autoantigen which was detected by sera from both patient B and C and degraded by C1 has been indicated (arrow). A 40-kDa autoantigen detected by serum from patient D was also degraded by C1 (arrow). (**b**) Sera from patients E-H detected insignificant autoantigens in the apoptotic U937 supernatant. In this validating experiment, serum from patient C was used as a positive control. A single blot was cut into 5 strips and incubated separately with the patients’ sera. Apoptotic cells: AC.

Autoantigens were detected strongly in the apoptotic supernatant by four patients’ sera (A-D) (Figure 6a) whilst four other patients’ sera (E-H) revealed faint autoantigens bands on the blots (Figure 6b). Sera from patient B and C detected similar autoantigen profiles while the patterns obtained with sera from patient A and D were distinct (Figure 6a). Two autoantigens were found degraded by C1. Detected by sera from patient B and C, an autoantigen of approximately 45-kDa diminished after C1 incubation and a new protein species of approximately 40-kDa appeared (Figure 6a). Another 40-kDa autoantigen was detected by the serum of patient D and this antigen diminished after C1 incubation. In this case, a new protein band of approximately 37-kDa appeared. The global profile of autoantigens in SLE patients reveals specific nature of C1 proteolytic activity, suggesting a mechanistic undertone in its targeting.

In summary, multiple lines of evidence suggest a selective range of non-complement substrates for the C1 proteases including both extracellular and intracellular proteins. We recently provided the first evidence that C1 cleaved two nuclear proteins NPM1 and nucleolin.^34^ In the present study, we provide new data that C1s cleaves the nuclear alarmin HMGB1 and impairs its ability to synergize with LPS in activating monocytes, macrophages and DC. This may help to explain why C1s deficiency causes SLE-like conditions.

## Discussion

The complement system consists of more than 30 proteins and can be activated through three pathways.^13,29^ Inherited deficiency for many of the complement proteins has been described which mostly present with increased susceptibility to microbial infections. However, deficiencies in C1q, C1r/C1s and C4, in addition to increasing the susceptibility to infection, are strongly associated with antinuclear autoimmunity individually.^29,51^ C1q protective role against autoimmunity revolves around its function in the clearance of apoptotic cellular debris and suppression of immune responses.^14,15,17–20,52^ For C4, its role in maintaining B cell tolerance was demonstrated using C4^−/−^ mice; these mice displayed increased germinal center numbers and reactions with anti-nucleolus autoreactive B cells.^53^

There is a critically unmet need to elucidate the protective mechanism of C1s against SLE that cannot be explained by C1s classical role in cleaving the complement protein C4 and C2 leading to the propagation of the complement cascade. Attribution of C1s protective role solely to the generation of downstream opsonins for apoptotic cells clearance is inadequate as deficiency of the downstream opsonic C3 component, produced from the action of C3 convertase (C4bC2a), lacked similar association with SLE.^29,51^ Hence, we attempt to distill the involvement and mechanism of C1s proteolytic role and contribution to programmed cell death.

C1s and other complement proteases are highly substrate-specific within the complement cascade, eg, C1s only cleaves C4, C2 and C1-INH. This ensures directional amplification of the cascade so as to activate the complement proteins in an orderly and proportional manner. Outside the complement system, growing evidences however suggest that C1s cleaves a broader spectrum of non-complement proteins^30–32^ and cleavage of intracellular proteins was initially suggested through bioinformatics prediction.^33^ We recently demonstrated that C1q gained access and bind to nucleolus to cause C1s activation and cleavage of nucleolar proteins during apoptosis.^34^ In this study, we demonstrated C1s cleavage of the alarmin protein HMGB1 and other intracellular proteins released by apoptotic cells.

Besides HMGB1, C1 also cleaved other proteins in the apoptotic supernatants including NPM1 and other autoantigens. However, majority of the proteins, including *β*-actin, HSP90*α*, RPLP0 and many autoantigens detected using patients’ sera (Figures 5 and 6), were not cleaved, demonstrating C1 proteases selectivity. Overall, C1 proteases or C1s can potentially cleave many intracellular proteins released during cell apoptosis and this could significantly reduce the immunogenicity of this cellular debris.

In this study, we demonstrated that HMGB1 synergism with suboptimal LPS to activate monocytes, macrophages and DC was effectively abolished with C1s digestion. Subclinical levels of LPS are commonly detected in the plasma especially in chronic diseases^54^ which can suppress T cell proliferation and retard monocyte response to further challenges with TLR ligands including LPS.^45^ A surge of HMGB1 could render otherwise subclinical levels of LPS inflammatory and injurious and C1s might have a role in regulating this.

The ability of C1q to bind to apoptotic cells and enhance their clearance via the process of efferocytosis has been well-defined.^14–17,52^ *In vivo*, C1q exists as a complex with C1r and C1s. The ability of these proteases to degrade intracellular proteins exposed by apoptotic cells when bound by C1q, adds another dimension of control in tissue scavenging where the proteolytic cleavage of alarmins and autoantigens can help complement the ‘immunological silent’ manner of efferocytosis.^16^ Cell apoptosis is intrinsically associated with the degradation of many proteins including autoantigens.^55^ C1 complex may therefore offer an additional mechanism that `complement’ intracellular proteases for the cleavage of cellular proteins. Impairment in this external proteolytic mechanism may therefore leave apoptotic cells with increased levels of alarmins and autoantigens and render the debris globally more immunogenic for autoreactive lymphocyte activation.

In conclusion, we provide evidence that C1s can cleave HMGB1, a nuclear alarmin, and other autoantigens released during cell apoptosis. Functionally, the cleavage of rHMGB1 abrogates its ability to synergize with LPS in inducing cytokines production in immune cells. This destruction of HMGB1 pro-inflammatory activity may partially help to explain C1s protective role against SLE.

## Materials and Methods

### Reagents

Purified sC1s, C1 complex, and C1-INH were purchased from Calbiochem (Billerica, MA, USA). RC1s and rHMGB1, both produced in mouse myeloma cells, were obtained from R&D systems (Minneapolis, MN, USA). His-HMGB1 produced in bacteria was obtained from GenScript, Co. (Piscataway, NJ, USA). LPS (*Escherichia coli,* serotype 055:B5) and mouse anti-NPM1 (clone FC82291) and anti-*β*-actin monoclonal antibodies (clone AC-74) were purchased from Sigma-Aldrich, Co. (St. Louis, MO, USA). A rabbit anti-HMGB1 antibody was obtained from Upstate Biotech (Billerica, MA, USA). Rabbit anti-C1s and goat anti-C1q antibodies were obtained from Quidel, Co. (San Diego, CA, USA). Rabbit antibodies for HSP90*α* and RPLP0 were obtained from Abcam plc (Cambridge, UK).

### Cell cultures

The Jurkat lymphoblast cells (ATCC) were cultured in RPMI1640 (Life Technologies, Carlsbad, CA, USA) supplemented with 10% (v/v) fetal calf serum (Thermo Scientific (HyClone), Waltham, MA, USA), 100 units/ml penicillin and 100 *μ*g/ml streptomycin, 2 mM l-glutamine, 1 mM sodium pyruvate and 0.0012% (v/v) *β*-mercaptoethanol. The U937 promonocytic cells (ATCC) were cultured in RPMI1640 with 10% (v/v) fetal calf serum, 100 units/ml penicillin, 100 *μ*g/ml streptomycin, and 2 mM l-glutamine. Peripheral blood mononuclear cells were isolated from buffy coats, provided by the Singapore National University Hospital Blood Donation Centre with Institutional approval, as described previously.^56^ Monocytes were isolated and, where macrophages and DC were required, cells were cultured for 6 days in RPMI1640, supplemented with 10% (v/v) bovine calf serum (HyClone), 100 units/ml penicillin and 100 *μ*g/ml streptomycin, 2 mM l-glutamine, 1 mM sodium pyruvate, and 0.0012% (v/v) *β*-mercaptoethanol. M-CSF (20 ng/ml) was replenished every two days to generate macrophages. GM-CSF (20 ng/ml) and IL-4 (40 ng/ml) were used to generate DC.

### Patient recruitment

Juvenile rheumatological patients were recruited from KK Women’s and Children’s Hospital with Institutional Review Board approval. Clinical data were collected blind to laboratory researchers (Supplementary Table 2). Blood (2 to 5 ml) was collected with consent into the Vacutainer Plus serum tubes (BD Biosciences, Franklin Lakes, NJ, USA) and, after clotting for 30 min, sera were harvested and stored at −80 °C.

### Bioinformatics

The Web-based PoPS software (http://pops.csse.monash.edu.au/) was used to predict C1s cleavage sites on HMGB1.^33,39^ The C1s cleavage site encompassed eight residues with a core arginine residue recognized by the S1 subsite (Figure 1a). A C1s cleavage site prediction model was constructed, based on published amino acid frequencies of C1s-cleavable octameric peptides identified in a phage display library,^33^ with previously described methodology (Supplementary Table 3).^57,58^ Predicted cleavage sites were scored and secondary or tertiary structures around them analyzed.^39^

### HMGB1 digestion and leukocyte stimulation

RHMGB1, His-HMGB1 and, as controls, BSA and complement C4, were treated with sC1s or C1 complex in 50 mM Tris, 150 mM NaCl, and 0.2% (w/v) polyethylene glycol 8000. Subsequent analysis was by SDS-PAGE or western blotting.

For leukocyte stimulation, rHMGB1 (5 *μ*g/ml) was digested with rC1s (2.75 *μ*g/ml) in 50 *μ*l reactions at 37 °C for 20 h. LPS was included (12.5–500 ng/ml) in some conditions. As controls, rC1s or rHMGB1 were separately incubated with LPS. Monocytes, macrophages and DC were re-suspended at 6×10^5^/ml in OPTI-MEM I (Life Technologies, Carlsbad, CA) supplemented with antibiotics and cultured in triplicate in 96-well plates (0.1 ml/well). The stimuli were added (2 *μ*l/well), and after 24 h, IL-6 and TNF-*α* were assayed in the culture media using the OptiEIA enzyme-linked immunosorbent assay (ELISA) kits (BD Biosciences, Franklin Lakes, NJ, USA).

### Generation of apoptotic cells

Jurkat cells were harvested and, after washing, re-suspended to 3×10^6^/ml in serum-free RPMI1640. In 6-well plates (2 ml/well), cells were UV-irradiated at 500 mJ/cm^2^ (Spectroline Select XLE-1000 UV crosslinker fitted with 254 nm lamps). Apoptosis was assessed using the apoptotic DNA ladder detection kit (BioVision, Milpitas, CA, USA). U937 cell apoptosis was similarly induced and after 24 h, the supernatant as a source of autoantigens for immunoblotting with patient sera was collected by centrifugation for 5 min at 500×*g*.^7^

### Cleavage of apoptotic cell antigens with C1 and C1s

UV-irradiated Jurkat cells were incubated for 2.5 h at 37 °C and 5% CO_2_ before being transferred to 1.5-ml tubes (490 *μ*l/tube) to which 10.4 *μ*l of C1 (240 *μ*g/ml), sC1s (240, 120 or 48 *μ*g/ml) or PBS was added. The supernatants were collected after 30 min incubation at 37 °C by centrifugation (5 min at 1000 g) and examined for protein cleavage (HMGB1, NPM1, HSP90, RPLP0 and *β*-actin) by western blotting. Where C1-INH was used, it was added at 105 *μ*g/ml.

### C1 cleavage of autoantigens

UV-irradiated U937 cells supernatant was treated with 48 *μ*g/ml of C1 in a 50-*μ*l reaction volume. After incubation for 1 h at 37 °C, the reactions were subjected to SDS-PAGE on 12.5% (w/v) gels and analyzed by western blotting with overnight incubation with rheumatological patients’ sera (1 : 5000 dilutions) and then horseradish peroxidase-conjugated goat anti-human IgG Fc (1 : 20 000 dilutions; Pierce, Rockford, IL, USA). Signals were visualized using the MPS ChemiDoc imaging system (Bio-Rad Lab., Hercules, CA, USA).

### SDS-PAGE and western blotting

Samples were reduced by heating in the presence of dithiothreitol (10 mM) and separated on 12.5% (w/v) gels. After electrotransfering, blots were first blocked for 1 h with 5% (w/v) non-fat milk in TBST (50 mM Tris, 150 mM NaCl, and 0.1% (v/v) Tween 20, pH 7.4) and then incubated overnight with specific antibodies. The blots were washed and incubated for 1 h with horseradish peroxidase-conjugated secondary antibodies. Signals were visualized using X-ray films or the Bio-Rad MPS ChemiDoc imaging system. Densitometry analysis was performed with the Image J software (version 1.43 u).^59^ Gels were also Commassie blue-stained^46^ or silver-stained.^60^

### Mass spectrometry

UV-irradiated Jurkat cells were cultured for 2.5 h and then treated for 30 min at 37 °C with C1 (5 *μ*g/ml) or sC1s (1 *μ*g/ml). After centrifugation for 5 min at 1000 g, supernatants were subjected to SDS-PAGE on 8–16% (w/v) gradient gels (Pierce). Samples were heated for 10 min at 100 °C in the presence of dithiothreitol (10 mM) without dye and alkylated for 30 min at room temperature with iodoacetamide (20 mM) in the dark. The gel was stained and bands were selectively excised based on their reduction or disappearance after C1 treatment. The gel slices were trypsin-digested, extracted and analyzed by liquid chromatography tandem mass spectrometry (Experimental Therapeutics Centre, Biopolis Shared Facilities, A-Star, Singapore). Data were analyzed and presented using the Scaffold_4.0.5 software (Proteome Software, Inc., Portland, OR, USA).

### Statistics

Data were expressed as mean values of experimental triplicates with standard deviation. Test for statistical significance was performed using the Student’s two-tailed unpaired *t*-test and *P*<0.05 was considered significant.

## Abbreviations

BSA: bovine serum albumin
C1-INH: C1-inhibitor
DC: dendritic cells
dsDNA: double-stranded DNA
HSP90*α*: heat shock protein 90 *α*
HMGB1: high-mobility group box 1
IGFBP5: insulin-like growth factor binding protein 5
IFN-*α*: interferon-*α*
LPS: lipopolysaccharide
LPR6: low-density lipoprotein receptor-related protein 6
NPM1: nucleophosmin 1
PBS: phosphate-buffered saline
His-HMGB1: polyhistidine-tagged HMGB1
PoPS: prediction of protease specificity
rC1s: recombinant C1s
rHMGB1: recombinant HMGB1
sC1s: serum C1s
SLE: systemic lupus erythematosus
TLR4: toll-like receptor 4
TNF-*α*: tumor necrosis factor-*α*
UV: ultra violet
RPLP0: 60S acidic ribosomal protein P0.
