# Supplementary material for: Proteolytic inactivation of nuclear alarmin high-mobility group box 1 by complement protease C1s during apoptosis
Source: Cell Death Discov. 2016 Sep 12;2:16069–. doi: 10.1038/cddiscovery.2016.69 (PMC5018544; doi:10.1038/cddiscovery.2016.69)
Supplement: Supplementary Tables [file cddiscovery201669-s1.doc]

**­­­Supplementary Tables Legends**

**Table S1: Potential C1s substrates identified by mass spectrometry.** Treatment of apoptotic Jurkat cells resulted in the loss or decrease in four band regions (A, B, C and D) that were visible on sodium dodecyl sulfate polyacrylamide gel electrophoresis (SDS-PAGE) gel by Commassie blue staining (Figure 5A). These corresponding band regions in untreated apoptotic supernatant were excised and analyzed by mass spectrometry. Proteins with identification probabilities lower than 100% had been excluded from this table. Multiple proteins were identified in each band region, which requires validation by Western blotting.

**Table S2: Clinical and laboratory characteristics of patients.** Sera of patients with rheumatological diseases were used to identify autoantigens in apoptotic U937 cell supernatant (Figure 6). Disease activities of the patients were scored according to SLEDAI-2K (SLE Disease Activity Index 2000) and a higher score is indicative of greater disease activity. Enzyme linked immunosorbent assay kits were used to determine autoantibody profiles for C1q (Orgentec Diagnostika GmbH, Mainz, Germany), dsDNA and nucleosomes (Euroimmun AG, Lübeck, Germany). The normal complement C3 and C4 levels are 0.82-1.85 g/L and 0.15-0.53 g/L respectively. ESR: erythrocytes sedimentation rate; SLE: Systemic Lupus Erythematosus; `-’: not determined.

**Table S3: C1s protease substrate prediction model.** A model was constructed based on the PoPS software and experimental data obtained from C1s-cleavable octameric peptides identified from a random phage display library. The relative weights (wt.) of each amino acid within each subsite and between different subsites were determined as previously described.^1^ First, amino acid frequency (n) at each subsite was derived from data previous published.^2^ A frequency of 5 or less was assigned the value `0’ and the wt. of each amino acid was calculated from its Δσ. Δσ = (Obs(χ)-nP(χ))/√( nP(χ)[1-P(χ)]) where n = 93 (the total number of C1s-cleavable peptide analyzed in the study by Kerr et al.^2^), Obs(χ) = observed frequency among the 93 peptides of an amino acid at one subsite, and P(χ) = number of allowed synonymous codons encoding the amino acid ÷ 31 (There are a total of 31 codons encoding the 20 amino acids). Δσ reflected the difference between the observed frequency Obs(χ) and expected frequency P(χ) in terms of standard deviation. When Δσ value is greater than 1, it means that the particular amino acid is over-represented at the particular subsite. Negative values were assigned the value `0’ meaning lack of contribution to C1s cleavage. At each subsite, an amino acid residue with the highest Δσ was assigned the value `5’ and the wt. of the other amino acids was scaled accordingly between 0 and 5. Lastly, the wt. of each subsite was obtained by dividing the highest Δσ value of the subsite with that of the arginine residue at the S1 subsite. Both three and one letter codes are used for the amino acid residues. ‘#’ denotes amino acid that would prevent C1s cleavage.

**Supplementary Tables**

**Table S1**

| **Bands** | **Identified Proteins** | **Molecular weight (kDa)** |
| --- | --- | --- |
| A | 26S proteasome non-ATPase regulatory subunit 2 | 100 |
|  | Isoform 2 of Heat shock protein HSP 90-alpha (HSP90a) | 98 |
|  | Importin subunit beta-1 | 97 |
|  | Elongation factor 2 | 95 |
|  | Transitional endoplasmic reticulum ATPase | 89 |
| B | T-complex protein 1 subunit alpha | 60 |
|  | T-complex protein 1 subunit eta | 59 |
|  | Isoform M2 of Pyruvate kinase isozymes M1/M2 | 58 |
|  | T-complex protein 1 subunit delta | 58 |
|  | T-complex protein 1 subunit zeta | 58 |
|  | T-complex protein 1 gamma isoform C | 56 |
| C | cDNA FLJ56389, highly similar to Elongation factor 1-gamma | 56 |
|  | Elongation factor 1-alpha 1 | 50 |
|  | Tubulin alpha-1C chain | 50 |
|  | Proteasome 26S non-ATPase subunit 11 variant (Fragment) | 48 |
|  | 60S ribosomal protein L4 | 48 |
|  | Isoform alpha-enolase of Alpha-enolase | 47 |
|  | Hsc70-interacting protein | 41 |
| D | Actin, cytoplasmic 1 | 42 |
|  | Transaldolase | 38 |
|  | Glyceraldehyde-3-phosphate dehydrogenase | 36 |
|  | 60S acidic ribosomal protein P0 (RPLP0) | 34 |
|  | Isoform 2 of Nucleophosmin (NPM1) | 29 |

Table S2

| **Patients** | **Rheumatological Disease** | **Flare Manifestations** | **SLEDAI-2K**  **(0 -105)** | **Laboratory Investigations** | | | | | |
| --- | --- | --- | --- | --- | --- | --- | --- | --- | --- |
|  |  |  |  | **ESR (mm/hr)** | **C3 (g/L)** | **C4 (g/L)** | **anti-dsDNA (IU/ml)** | **anti-nucleosome (disease cut-off**  **>20 RU/ml)** | **Anti-C1q (disease cut-off**  **>10 U/ml)** |
| A | SLE | Hematological, discoid rash, arthritis, serositis, and glomerulonephritis | 23 | 70 | 0.13 | 0.01 | 290 | 232 | 79 |
| B | Undifferentiated connective tissue disease (Cutaneous vasculitis) | - | - | - | - | - | - | - | - |
| C | Sjogren’s syndrome (inactive disease) | - | - | - | - | - | - | - | - |
| D | SLE | Rash | 2 | 1 | 0.73 | 0.08 | 101 | - | - |
| E | SLE | Gastrointestinal vasculitis, and glomerulonephritis | 27 | 50 | 0.34 | 0.01 | 263 | 194 | 87 |
| F | SLE | - | 0 | 55 | 0.63 | 0.02 | 221 | 147 | 10 |
| G | SLE | - | 0 | 5 | 0.74 | 0.19 | 0 | 3 | 15 |
| H | SLE | Malar rash, and lymphopenia | 2 | 20 | 0.48 | 0.14 | 280 | 206 | 11 |

Table S3

| **Subsites** | **S4** | | | | **S3** | | | | **S2** | | | **S1** | | **S1'** | | | **S2'** | | | | **S3'** | | | **S4'** | | |
| --- | --- | --- | --- | --- | --- | --- | --- | --- | --- | --- | --- | --- | --- | --- | --- | --- | --- | --- | --- | --- | --- | --- | --- | --- | --- | --- |
| **Wt.** | **1.49** | | | | **1.67** | | | | **1.86** | | | **5** | | **0.70** | | | **0.77** | | | | **0.43** | | | **0.56** | | |
| **Amino acids** | **n** | **Δσ** | **Wt.** | **n** | | **Δσ** | **Wt.** | **n** | | **Δσ** | **Wt.** | | **Wt.** | **n** | **Δσ** | **Wt.** | **n** | **Δσ** | **Wt.** | **n** | | **Δσ** | **Wt.** | **n** | **Δσ** | **Wt.** |
| Gly, G | 8 | 0.84 | 0.48 | 14 | | 3.38 | 1.72 | 32 | | 10.97 | 5.00 | | # | **-** | **-** | 0.00 | 11 | 2.11 | 2.31 | 10 | | 1.69 | 3.33 | **-** | **-** | 0.00 |
| Ala, A | 14 | 3.38 | 1.92 | 7 | | 0.42 | 0.21 | 12 | | 2.53 | 1.15 | | # | 12 | 2.53 | 3.08 | 10 | 1.69 | 1.85 | - | | - | 0.00 | 10 | 1.69 | 2.86 |
| Val, V | 8 | 0.84 | 0.48 | 12 | | 2.53 | 1.29 | - | | - | 0.00 | | # | 12 | 2.53 | 3.08 | - | - | 0.00 | 12 | | 2.53 | 5.00 | 13 | 2.95 | 5.00 |
| Leu, L | 8 | -0.35 | 0.00 | 37 | | 9.82 | 5.00 | 6 | | -1.05 | 0.00 | | # | 13 | 1.40 | 1.71 | 22 | 4.56 | 5.00 | 11 | | 0.70 | 1.38 | 10 | 0.35 | 0.59 |
| Ile, I | - | - | 0.00 | - | | - | 0.00 | - | | - | 0.00 | | # | 10 | 4.11 | 5.00 | 8 | 2.93 | 3.22 | - | | - | 0.00 | - | - | 0.00 |
| Pro, P | - | - | 0.00 | - | | - | 0.00 | - | | - | 0.00 | | # | - | - | 0.00 | - | - | 0.00 | - | | - | 0.00 | - | - | 0.00 |
| Phe, F | 7 | 2.35 | 1.33 | - | | - | 0.00 | - | | - | 0.00 | | # | - | - | 0.00 | - | - | 0.00 | - | | - | 0.00 | - | - | 0.00 |
| Tyr, Y | 18 | 8.80 | 5.00 | - | | - | 0.00 | 6 | | 1.76 | 0.80 | | # | - | - | 0.00 | - | - | 0.00 | - | | - | 0.00 | - | - | 0.00 |
| Trp, W | - | - | 0.00 | - | | - | 0.00 | - | | - | 0.00 | | # | - | - | 0.00 | - | - | 0.00 | - | | - | 0.00 | 6 | 1.76 | 2.98 |
| Ser, S | 15 | 2.10 | 1.20 | - | | - | 0.00 | - | | - | 0.00 | | # | 16 | 2.46 | 2.99 | - | - | 0.00 | - | | - | 0.00 | - | - | 0.00 |
| Thr, T | - | - | 0.00 | - | | - | 0.00 | - | | - | 0.00 | | # | - | - | 0.00 | - | - | 0.00 | - | | - | 0.00 | - | - | 0.00 |
| Cys, C | - | - | 0.00 | - | | - | 0.00 | - | | - | 0.00 | | # | - | - | 0.00 | - | - | 0.00 | - | | - | 0.00 | - | - | 0.00 |
| Met, M | - | - | 0.00 | - | | - | 0.00 | - | | - | 0.00 | | # | - | - | 0.00 | - | - | 0.00 | - | | - | 0.00 | - | - | 0.00 |
| Asn, N | - | - | 0.00 | - | | - | 0.00 | - | | - | 0.00 | | # | - | - | 0.00 | - | - | 0.00 | - | | - | 0.00 | - | - | 0.00 |
| Gln, Q | - | - | 0.00 | - | | - | 0.00 | - | | - | 0.00 | | # | - | - | 0.00 | - | - | 0.00 | - | | - | 0.00 | - | - | 0.00 |
| Asp, D | - | - | 0.00 | - | | - | 0.00 | - | | - | 0.00 | | # | - | - | 0.00 | - | - | 0.00 | - | | - | 0.00 | - | - | 0.00 |
| Glu, E | - | - | 0.00 | - | | - | 0.00 | - | | - | 0.00 | | # | - | - | 0.00 | - | - | 0.00 | - | | - | 0.00 | - | - | 0.00 |
| Lys, K | - | - | 0.00 | - | | - | 0.00 | - | | - | 0.00 | | # | - | - | 0.00 | - | - | 0.00 | - | | - | 0.00 | - | - | 0.00 |
| Arg, R | - | - | 0.00 | - | | - | 0.00 | - | | - | 0.00 | | 5 | 8 | -0.35 | 0.00 | 10 | 0.35 | 0.38 | 15 | | 2.10 | 4.15 | - | - | 0.00 |
| His, H | - | - | 0.00 | - | | - | 0.00 | - | | - | 0.00 | | # | - | - | 0.00 | - | - | 0.00 | - | | - | 0.00 | - | - | 0.00 |

**References**

1. Song J, Matthews AY, Reboul CF, Kaiserman D, Pike RN, Bird PI*, et al.* Predicting serpin/protease interactions. *Methods Enzymol* 2011, **501:** 237-273.

2. Kerr FK, O'Brien G, Quinsey NS, Whisstock JC, Boyd S, de la Banda MG*, et al.* Elucidation of the substrate specificity of the C1s protease of the classical complement pathway. *J Biol Chem* 2005, **280**(47)**:** 39510-39514.
